# EBV-Associated Gastric Cancer; An In Situ Hybridization Assay on Tissue Microarray: A Multi-Region Study from Four Major Provinces of Iran

**DOI:** 10.34172/aim.2024.28

**Published:** 2024-04-01

**Authors:** Maryam Abolhasani, Ata ollah Mohseni, Ramin Shakeri, Ali Khavanin, Mehrdad Khajehei, Abbasali Omidi, Bita Geramizadeh, Ensieh Shafigh, Farshad Naghshvar, Payam Fathizadeh, Leyla Taghizadehgan, Atoosa Gharib, Margaret L. Gulley, Sanford M. Dawsey, Reza Malekzadeh, Charles S. Rabkin, Mohammad Vasei

**Affiliations:** ^1^Oncopathology Research Center, School of Medicine, Iran University of Medical Sciences, Tehran, Iran; ^2^Dr Mohseni’s Pathobiology Laboratory, Nour, Iran; ^3^Digestive Oncology Research Center, Digestive Disease Research Institute, Tehran University of Medical Sciences, Tehran, Iran; ^4^Emergency Medicine Department, Ahvaz Jundishapur University of Medical Sciences, Ahvaz, Iran; ^5^Shiraz Medical School, Shiraz University of Medical Sciences, Shiraz, Iran; ^6^Department of Pathology, Mashhad University of Medical Sciences, Mashhad, Iran; ^7^Department of Pathology, Transplantation Research Center, Shiraz University of Medical Sciences, Shiraz, Iran; ^8^Department of Pathology, Babol University of Medical Sciences, Babol, Iran; ^9^Department of Pathology, Mazandaran University of Medical Sciences, Sari, Iran; ^10^Department of Pathology and Laboratory Medicine, Apadana Hospital, Ahvaz, Iran; ^11^Taghizadegan’s Pathology and Laboratory Medicine, Shiraz, Iran; ^12^Department of Pathology, Modarres Hospital, Shahid Beheshti University of Medical Sciences, Tehran, Iran; ^13^Department of Pathology, University of North Carolina, Chapel Hill, NC, USA; ^14^Metabolic Epidemiology Branch, Division of Cancer Epidemiology and Genetics, National Cancer Institute, Rockville, MD, USA; ^15^Infections and Immunoepidemiology Branch, Division of Cancer Epidemiology and Genetics, National Cancer Institute, Rockville, MD, USA; ^16^Gene Therapy Research Center, Digestive Disease Research Institute, Tehran University of Medical Sciences, Tehran, Iran

**Keywords:** Gastric cancer, Epstein-Barr virus, Iran, Tissue microarray

## Abstract

**Background:**

Gastric cancer is the fourth leading cause of cancer-related deaths in the world. The identification of gastric cancer subtypes related to recognizable microbial agents may play a pivotal role in the targeted prevention and treatment of this cancer. The current study is conducted to define the frequency of Epstein-Barr virus (EBV) infection in gastric cancers of four major provinces, with different incidence rates of gastric cancers, in Iran.

**Methods:**

Paraffin blocks of 682 cases of various types of gastric cancer from Tehran, South and North areas of Iran were collected. Twelve tissue microarray (TMA) blocks were constructed from these blocks. Localization of EBV in tumors was assessed by in situ hybridization (ISH) for EBV-encoded RNA (EBER). Chi-squared test was used to evaluate the statistical significance between EBV-associated gastric cancer (EBVaGC) and clinicopathologic tumor characteristics.

**Results:**

Fourteen out of 682 cases (2.1%) of gastric adenocarcinoma were EBER-positive. EBER was positive in 8 out of 22 (36.4%) of medullary carcinomas and 6 out of 660 (0.9%) of non-medullary type, which was a statistically significant difference (*P*<0.001). The EBVaGCs were more frequent in younger age (*P*=0.009) and also showed a trend toward the lower stage of the tumor (*P*=0.075).

**Conclusion:**

EBV-associated gastric adenocarcinoma has a low prevalence in Iran. This finding can be due to epidemiologic differences in risk factors and exposures, and the low number of gastric medullary carcinomas in the population. It may also be related to gastric tumor heterogeneity not detected with the TMA technique.

## Introduction

 The development of gastric cancer is a multistep and multifactorial process. The 2010 WHO classification categorizes gastric cancers into four major histologic types: tubular, papillary, mucinous and poorly cohesive (including signet ring cell carcinoma) and also includes some uncommon histologic variants,^1^ but there are two commonly used classes of gastric cancer according to Lauren’s classification; intestinal and diffuse type.^2^ The intestinal type is often related to environmental factors such as Helicobacter pylori infection, nitrate containing diet, and high salt intake,^3^ and precursor lesions include intestinal metaplasia and gastric atrophy.^4^ The diffuse type is associated with certain genetic abnormalities such as loss of CDH1 (Cadherin 1), but there is no clear association with environmental factors.^5,6^

 After dietary factors and tobacco smoking, infectious diseases represent the third most common cause of cancer worldwide.^7^ Chronic infection with Helicobacter pylori, human papillomaviruses (HPV), hepatitis B (HBV) and C (HCV) viruses are each responsible for approximately 5% of all human cancers and, altogether, they account for the about 15% of cancers worldwide.^8,9^ Epstein-Barr virus (EBV), as well as some types of HPV, KSHV, HBV, HCV, HTLV-I, and HIV-1, are designated as carcinogenic to humans (Group 1) by International Agency for Research on Cancer (IARC).^7^

 EBV is associated with or implicated in the etiology of several lymphoid tumors such as Burkitt’s lymphoma, Hodgkin’s lymphoma, and nasal NK/T cell lymphoma. It has also been detected in the tumor cells of some epithelial neoplasms, particularly those with lymphoepithelioma-like histology in the nasopharynx and lymphoepithelioma-like or medullary type adenocarcinoma.^10^

 Recently, the Cancer Genome Atlas (TCGA) network classified gastric adenocarcinoma into four major subtypes: (1) tumors positive for Epstein-Barr virus, which often display recurrent PIK3CA mutation, DNA hypermethylation, and amplification of JAK2 and the genes encoding PD-L1 and PD-L2; (2) microsatellite unstable tumors, which show elevated mutation rates including in genes encoding targetable oncogenic signaling proteins; (3) genomically stable tumors, which are enriched for the diffuse histological variant and mutations of RHOA or fusions involving RHO-family GTPase-activating proteins; and (4) tumors with chromosomal instability, which have TP53 mutation, marked aneuploidy, and focal amplification of receptor tyrosine kinases.^11^

 As many as 80% of gastric carcinomas with lymphoid stroma have been reported to be EBV-associated.^12,13^ Carcinoma with lymphoid stroma (medullary carcinoma) is one of the uncommon histologic subtypes of gastric cancer which has a well-defined margin and is characterized by nests or sheets of neoplastic cells with a non-desmoplastic stroma having a prominent lymphoid infiltrate. This tumor is defined by dense lymphocytic infiltration, and the number of tumor-infiltrating lymphocytes is greater than that the number of malignant cells, with a syncytial growth pattern of tumor cells with indistinct cytoplasmic borders and poorly formed glandular structures. It occurs mostly in the proximal stomach and has a more favorable clinical outcome.^12^ Interestingly, EBV is only recognized in the malignant and dysplastic cells but not in non-neoplastic epithelial cells.^14^ Bortezomib, a proteasome inhibitor, can target infected tumor cells and induce EBV kinase and, in turn, make infected cells more susceptible to killing by other drugs, so the presence of EBV in gastric cancer may give some hope for targeted therapy.^15^ Checkpoint inhibitor therapy is also reported to be effective in EBV-associated gastric cancer (EBVaGC).^16^

 EBV has been investigated in gastric cancer in all 5 continents, including the USA, Chile, Brazil, Mexico, Peru, Colombia, Japan, Hong Kong, Taiwan, Korea, China, India, Malaysia, Pakistan, Kazakhstan,^17^ Tunisia,^18,19^ and Papua New Guinea.^20^

 EBVaGC is estimated to constitute about 8% to 10% of all gastric cancers, with a relatively similar prevalence in cases from Asia (8.3%), Europe (9.2%), and the Americas (9.9%)^21^ Also, there are two studies reporting EBV positivity from Tunisia in Africa to range from 4.1% to 14.8%.^18,19^ The frequency of EBV-associated gastric carcinomas in Papua New Guinea in Oceania, assessed by EBER RNA expression, was 1.3%: the lowest ever reported in the world.^20^

 In Iran, gastric cancer is among the leading solid cancers. Although it occurs with variable incidence in different geographic regions of the country, the total incidence is increasing.^22^ Its incidence is slightly higher than that in the Middle East Cancer Consortium (MECC) or the United States, is similar to that in the United Kingdom, and is much lower than that in Japan, Korea and Southeast Asia.^21^

 There are few studies in Iran about the prevalence of EBV in gastric cancer.^23-27^ In these studies, the frequency of EBV-associated gastric carcinoma ranged from 3% in a research by Abdirad et al, which used an in situ hybridization (ISH) method,^23^ to 49.2% in a research by Fattahi et al, which was performed by polymerase chain reaction (PCR).^27^

 There is no large multicenter survey about the association of EBV with gastric cancer in Iran. The aim of the present study was to investigate the frequency of EBV in paraffin blocks of gastric carcinoma retrieved from 4 different geographic regions of Iran.

## Materials and Methods

###  Sample Selection

 Paraffin blocks of 682 cases of various types of gastric cancer from 7 different regions of the Islamic Republic of Iran were included in the study. Of these, 672 cases were resected gastric cancers provided from pathology departments of university hospitals in Shiraz, Mashad, Sari, Babol and Tehran and biopsies from ten additional cases were prepared from two centers in Tehran.

 Patient age and gender, the anatomical site of the tumor, histological classification according to Lauren’s classification and the 2010 WHO classification system, histological subtypes and pathological tumor stage were recorded. None of the patients had received chemotherapy or radiation therapy before surgery.

###  Ethics 

 Retrieval of tissue and clinical data was performed according to the regulations of the local ethics review board and the data safety laws in Iran.

###  Tissue Microarray

 To construct tissue microarray (TMA) blocks, hematoxylin and eosin (H&E)-stained slides were first reviewed by a pathologist. The most representative three regions of the tumor were marked on the slides. TMA blocks with one millimeter diameter cores were made, using an MTA-1 manual arrayer (Beecher Instruments, Sun Prairie, WI). Cores were harvested from donor blocks and transferred into a recipient block. Each TMA contained up to 62 duplicate cores from gastric cancers. Eleven blocks were made up of resected gastric adenocarcinomas; this means that each TMA contained up to 124 cores, including duplicate cores from each of up to 62 tumors. The 12th block contained 20 cores from 10 endoscopic biopsy cases of gastric adenocarcinoma collected from the Digestive Disease Research Institute (DDRI) archives in Tehran.

###  In Situ Hybridization

 ISH assay is the gold standard method for assigning EBV status in tumor tissues. ISH is a reliable method, but it requires invasive biopsy and complex techniques, rendering it inappropriate as a screening tool in a subpopulation at increased risk for EBVaGC.^28^ PCR methods are more sensitive but less specific than ISH.^28^ In one study, EBV was detected by PCR in 90.2% of stomach cancer cases, whereas EBER positivity localized to the malignant cells by ISH was found in just 11% of these tissues^29^

 In our study, EBER and RNA preservation control hybridizations were performed on paraffin sections of TMAs using EBER and oligo(dT) control probes (Universal ISH Detection Kit, Leica Biosystems, Berlin, Germany) and the manufacturer’s instructions on the Leica Biosystems Bond-III Automated IHC/ISH Stainer. EBER ISH was interpreted alongside the results of the RNA preservation control ISH. An H&E stain was evaluated to localize tumor, and presence of EBER signal in malignant cells was interpreted as EBER-positive, while lack of signal in malignant cells was judged EBER-negative when RNA was preserved, or EBER-uninterpretable when RNA preservation was lacking.

###  Statistics

 The data are presented as the mean ± standard deviation (SD) or as numbers and percentages. Chi-square test was used for the evaluation of the statistical association between gastric cancer EBV status and the clinicopathologic characteristics. *P* values of ≤ 0.05 were considered statistically significant. Statistical analyses were performed using SPSS for Windows, version 20.

## Results

 A total of 682 cases were included in the study. Of these, 269 patients (39.5%) were male, 74 patients (10.8%) were female and in 339 cases (49.7%), the gender status was missing. The mean age was 60.0 ± 24.2 years, the median age was 60.5 years, and the age range was 29–92.

 Using Lauren’s classification, 314 cases (46%) were intestinal type, 133 (19.6%) diffuse type, 108 (15.8%) mixed type, and the pathologic subtype of 127 cases (18.6%) was not available.

 According to the 2010 WHO classification, 218 (32%) were tubular, 37 (5.4%) papillary, 150 (22%) non cohesive, 22 (3.2%) mucinous, 18 (2.6%) rare variants and 108 (15.9%) mixed gastric carcinomas. The pathologic subtypes of 129 cases (18.9%) were not available. The total number of medullary carcinomas or carcinomas with lymphoid stroma in our study was 22 (3.2%).

 In this study, we included 160 (23.4%) well differentiated, 77 (11.3%) moderately differentiated, 300 (44%) poorly differentiated, and 2 (0.3%) undifferentiated gastric carcinomas. The histologic grade of 143 cases (21%) was not available. Considering stage, one case (0.2%) was pTis and 22 (3.2%), 5 (0.7%), 35 (5.1%), 416 (61%) and 26 (3.8%) cases were pT0, pT1, pT2, pT3 and pT4, respectively. In 177 cases (26%), the pT stage was not available.

 A total of 219 (32.1%) cases had no lymph node metastasis. Cases with pN1, pN2, pN3 amounted to 255 (37.4%), 76 (11.1%) and 17 (2.5%), respectively. The number of cases with unavailable pN was 115 (16.9%). Furthermore, 470 cases (68.9%) had no metastasis, 29 cases (4.3%) had distant metastasis and in 183 cases (26.8%), the status of metastasis was unknown.

 The location of tumor was cardia, body and antrum in 45 cases (6.6%), 8 (1.2%) and 89 (13%) respectively and in 540 cases (79.2%) the location was not mentioned.

 There were 682 evaluable tumors, including 14 (2.1%) that were EBER-positive by ISH and 668 that were EBER-negative. The mean age of EBER-positive cases was 59.3 ± 22.0 years, ranging from 34 to 70, and the median age was 62.5 year. Ten cases (71.4%) were male and in four cases (28.6%), the gender was not mentioned (Table 1).

**Table 1:** Distribution of Age, Sex and Histologic Subtypes in EBVaGC and non-EBVaGC

	**EBVaGC**	**Non-EBVaGC**	***P *** **value**
Total number, N (%)	14 (2.1)	668 (97.9)	
Gender, N (%)			
Male	10 (71.4)	259 (38.7)	0.315
Female		74 (11.1)	
Unknown	4 (28.6)	335 (50.2)	
Age (mean ± SD)	59.3 ± 22.0 years	60.1 ± 24.6 years	0.009
Histologic Subtypes – Lauren Classification, N (%)			
Intestinal	9 (64.3)	305 (45.7)	0.209
Diffuse	3 (21.4)	130 (19.5)	
Mixed		108 (16.1)	
Unknown	2 (14.3)	125 (18.7)	
Histologic Subtypes – 2010 WHO Classification, N (%)			
Tubular	7 (50.0)	211 (31.5)	0.310
Papillary		37 (5.6)	
Non-cohesive	4 (28.6)	146 (21.9)	
Mucinous		22 (3.3)	
Rare Variants	1 (7.1)	17 (2.5)	
Mixed		108 (16.2)	
Unknown	2 (14.3)	127 (19)	
Lymphoid Stroma (Medullary Features)			
Present	8 (57.1)	14 (2.1)	< 0.001
Absent	6 (42.9)	654 (97.9)	

 Using Lauren’s classification, in EBER-positive cases, nine cases (64.3%) were intestinal type and three (21.4%) diffuse type. The pathologic subtype of two cases (14.3%) was not available (Table 1).

 According to the 2010 WHO classification, in EBER-positive cases, seven (50%) were tubular, four (28.6%) were non-cohesive and one (7.1%) was another rare variant. The pathologic subtype of two cases (14.3%) was not available (Table 1). Eight (57.1%) of 14 EBER-positive were medullary carcinomas or carcinomas with lymphoid stroma.

 In EBER-positive cases, one (7.1%), nine (64.3%), and one (7.1%) were pT0, pT3, and pT4, respectively, while in three cases (21.5%), the pT classification was not available. Seven (50%), three (21.5%) and one (7%) were pN0, pN1, pN3, respectively, and in three cases (21.5%), the pN classification was not available. Nine cases (64.3%) had no metastasis. One case (7.1%) had a distant metastasis, and the status of metastasis of four cases (28.6%) was not available. One (7.1%), six (42.9%), four (28.7%), and one (7.1%) had a pathologic stage of 0, II, III and IV, respectively. In two cases (14.2%), the pathologic stage was not mentioned (Table 2).

**Table 2:** Distribution of Histologic Grade and Pathologic Stage in EBVaGC and Non-EBVaGC.

	**EBVaGC**	**Non-EBVaGC**	***P *** **Value**
Total number N (%)	14 (2.1)	668 (97.9)	
Grade N (%)			
Well differentiated	0	160 (24)	0.181
Moderately differentiated	2 (14.3)	75 (11.2)	
Poorly differentiated	9 (64.3)	291 (43.5)	
Undifferentiated		2 (0.3)	
Unknown	3 (21.4)	140 (21)	
pT N (%)			
pTis		1 (0.2)	0.216
pT0	1 (7.1)	21 (3.2)	
pT1		5 (0.7)	
pT2		35 (5.3)	
pT3	9 (64.3)	407 (60.9)	
pT4	1 (7.1)	25 (3.7)	
Unknown	3 (21.5)	174 (26%)	
pN N (%)			
pN0	7 (50)	212 (31.8)	0.576
pN1	3 (21.5)	252 (37.7)	
pN2		76 (11.3)	
pN3	1 (7)	16 (2.4)	
Unknown	3 (21.5)	112 (16.8)	
pT M (%)			
pM0	9 (64.3)	461 (69)	0.765
pM1	1 (7.1)	28 (4.2)	
Unknown	4 (28.6)	179 (26.8%)	
pTNM N (%)			
Stage 0	1 (7.1)	42 (6.3)	0.075
Stage 1		22 (3.3)	
Stage 2	6 (42.9)	123 (18.4)	
Stage 3	4 (28.7)	262 (39.2)	
Stage 4	1 (7.1)	44 (6.6)	
Unknown	2 (14.2)	175 (26.2)	
Location N (%)			
Cardia	0 (0)	45 (6.7)	0.554
Body	0 (0)	8 (1.2)	
Antrum	1 (7.1)	88 (13.2)	
Unknown	13 (92.9)	527 (78.9)	

 There were 668 EBER-negative patients, of whom 259 (38.7%) were male, 74 (11.1%) were female, and in 335 cases (50.2%), the gender was unavailable. The mean age was 60.1 ± 24.6 years, the median age was 60 years, and the age range was 29-92 (Table 1).

 Using Lauren’s classification, in EBER-negative cases, 305 cases (45.7%) were intestinal type, 130 (19.5%) diffuse type, 108 (16.1%) mixed type, and the pathologic subtype of 125 cases (18.7%) was not available (Table 1).

 According to the 2010 WHO classification, in EBER-negative cases, 211 (31.5%) were tubular, 37 (5.6%) papillary, 146 (21.9%) non-cohesive, 22 (3.3%) mucinous, 17 (2.5%) rare variants and 108 (16.2%) mixed gastric carcinomas. The pathologic subtype of 127 cases (19%) was not available (Table 1).

 Fourteen (63.6%) of the 22 medullary carcinomas or carcinomas with lymphoid stroma were EBER-negative.

 In EBER-negative cases, 160 (24%) were well differentiated, 75 (11.2%) moderately differentiated, 291 (43.5%) poorly differentiated, and 2 (0.3%) undifferentiated gastric carcinomas. The grade of 140 cases (21%) was unavailable (Table 2).

 In EBER-negative cases, one case (0.2%) was pTis and 21 (3.2%), 5 (0.7%), 35 (5.3%), 407 (60.9%) and 25 (3.7%) were pT0, pT1, pT2, pT3 and pT4, respectively. In 174 cases (26%), the pT classification was not available.

 In EBER-negative cases, 212 cases (31.8%) had no lymph node metastasis. Cases with pN1, pN2, and pN3 amounted to 252 (37.7%), 76 (11.3%) and 16 (2.4%), respectively. The number of cases with unavailable pN was 112 (16.8%). Moreover, 461 cases (69%) had no metastasis, 28 (4.2%) had distant metastasis and in 179 cases (26.8%), the status of metastasis was unknown. Overall, 42 (6.3%), 22 (3.3%), 123 (18.4%), 262 (39.2%), and 44 (6.6%) cases were in pathological stages 0, I, II, III and IV, respectively. In 175 (26.2%) cases, the pathologic stage was unavailable (Table 2).

 EBER was positive in 8 (36.4%) out of 22 gastric medullary carcinomas and 6 (0.9%) out of 660 non-medullary type gastric carcinomas, which was a statistically significant difference (*P* < 0.001). The EBVaGCs were also shown to be more frequent in younger age (*P* = 0.009) and also showed a trend toward lower pathologic stage (*P* = 0.075) although it was not statistically significant.

 There was no statistically significant difference between EBV-associated and EBV-negative gastric carcinomas regarding sex (*P* = 0.315), histologic subtypes according to Lauren’s classification (*P* = 0.209), histologic subtypes according to the 2010 WHO classification (*P* = 0.310), location of the tumor (cardia vs. non-cardia) (*P* = 0.554) or histologic grade (*P* = 0.181).

## Discussion

 The results of our study showed that 2.1% (14) of gastric adenocarcinoma were EBER-positive. EBER was positive in 8 out of 22 (36.4%) medullary carcinomas and 6 out of 660 (0.9%) non-medullary type, which showed a statistically significant difference (*P* < 0.001). The EBV associated gastric cancers were more frequent in younger age (*P* = 0.009) and also showed a trend toward lower stage (*P* = 0.075) although it was not statistically significant.

 The data in our research were collected from five cities and had some missing items. The whole H&E sections of cases from Tehran were available and reviewed by pathologists for subtyping of tumors and we had access to computer system for completing the demographic data, but we only had TMA paraffin embedded blocks of cases and collected data sheets of other provinces that were incomplete in some items. We could not use sections prepared from TMA for missing data of grading, subtyping or staging as whole H&E sections were needed for determining these items that were not available.

 Although the large sample size of our study could in part compensate for some missing data, we emphasize standard reporting of gastric cancer according to the International Collaboration for Cancer Reporting (ICCR), College of American Pathologists (CAP) or other approved standard protocols to provide necessary data for proper treatment of patients and future studies.

 According to data published by the national cancer registry of the ministry of health of Iran in 2014, the geographic distribution of gastric cancer varies in different provinces of Iran and is highest in Ardebil in northwestern Iran and decreases toward south with the lowest rate in Hormozgan. The provinces of Iran can be divided by into 4 categories according to the age-standardized incidence rate (ASR) of gastric cancer per 100 000 in men based on this data: Very high with ASR more than 30, high with ASR of 20-30, medium with ASR of 15–20 and low with ASR of less than 15.^30^

 Our study was performed on tissue and data collected from 4 provinces of Iran; thus, it cannot be generalized to the whole country and more centers from all over the country should be studied in future researches; nevertheless, it included provinces in all 4 categories of ASR of gastric cancer: Mashad in Khorasan-e-Razavi with very high incidence, Babol and Sari in Mazandaran with high incidence, Tehran in Tehran with medium incidence and Shiraz in Fars with low incidence.^30^

 EBVaGC is a non-endemic disease distributed throughout the world.^31,32^ However, there are some regional differences in the incidence of EBVaGC. The incidence of EBVaGC ranges from the highest (16%-18%) in the USA and Germany to the lowest in China (4.3%),^17,33^ Tunisia (4.1%),^18^ Peru (3.9%),^34^ and Papua New Guinea (1.3%).^20^ A Japanese study investigated the incidence of EBV-positive cases in gastric cancers in several areas. The study indicated that EBVaGC prevalence was inversely related to the gastric carcinoma incidence.^35^

 There are few studies about the frequency of EBVaGC in Iran. In a study by Abdirad et al, the presence of EBV-encoded small RNA-1 (EBER-1) was evaluated in 273 formalin fixed paraffin-embedded blocks of gastric carcinoma by ISH.^23^ They found EBV positivity in nine of the gastric carcinoma cases (3%), similar to our result of 2.1%. The proportion of EBV-positive cases in the diffuse histologic subtype was higher than in the intestinal subtype. EBV-positive cases had no relation with sex, age, location or invasion.

 In a study by Leila et al, 90 paraffin blocks of cases of gastric cancer were evaluated for the frequency of EBV and CMV viruses by real-time PCR for EBER and PP65 genomes for CMV. EBV status and CMV infections were positive in 10 (11.1%) and 7 (7.77%) cases, respectively. Although in this study, the EBV-positive status was more prevalent in males, there was no statistically significant association between EBV positivity in gastric cancer and sex or age.^24^

 In a study by Faghihloo et al, 90 formalin fixed paraffin-embedded blocks of gastric carcinoma from Iran were investigated for the presence of the EBV genome by quantitative real-time PCR. EBV was positive in six cases (7%). In this study, there was no significant association between EBV infection and sex, age, location or differentiation of the tumor.^26^

 In another Iranian research performed by Sarshari et al, EBER-ISH was studied in 68 gastric cancer biopsies.^25^ In their study, the prevalence of EBVaGC was 5.8% and lower among Iranian patients with gastric cancer than the world prevalence, which is concordant with the results of Abdirad et al,^23^ as well as ours.

 Fattahi et al showed different results for the frequency of EBV infection in gastric cancer in comparison with previous studies.^27^ In this study, 63 gastric adenocarcinomas and their adjacent non-tumor tissue, and 21 gastric tissues of healthy persons were assessed by PCR and real-time PCR assays for EBV, CMV, and HBV infections. Viral infection (EBV, CMV, or HBV) was identified in 39/63 (61.9%) and 1/21 (4.7%) of gastric cancer patients (tumoral or non-tumoral tissue) and healthy individuals, respectively, and EBV infection was the most frequent infection (49.2%). EBV DNA was found in 49.2% of tumor samples (31/63) and only in 4.7% (1/21) of normal tissues (*P* = 0.005). The viral load of EBV was higher in earlier grades and stages than advanced grades and stages, which is similar to our study, considering the trend of EBVaGC toward lower tumor stage.^26^

 In part, differences in prevalence can be attributed to the methods of detection of EBV in these studies. EBV DNA was first discovered by PCR in a paraffin-embedded blocks of a lymphoepithelioma-like gastric carcinoma.^36^ There are many studies that use ISH assays for localizing EBV in gastric carcinoma tissues.^29,37^ Very few studies have used whole-genome sequencing for EBV infection, although one study on 201 gastric cancers from Canada reported that 13 (6.5%) were EBV-positive,^38^ and the other one using The Cancer Genome Atlas found 26 EBV-positive gastric tumors out of 256 (9%) cases.^39^ Serological markers for EBV have been used to determine the cumulative lifetime exposure and reactivation of a viral infection, but serology is not used for evaluation of EBVaGCs.^28^

 In study by Li et al, 53 (12.6%) of 420 patients were positive for EBV by EBER ISH. In this study, EBVaGC was more common in males and associated to early T and TNM stages. This study is concordant with our study regarding the trend of EBVaGC toward lower stage.^40^

 In our study, we used ISH on TMA blocks to detect and localize EBV in gastric carcinomas. TMA is considered a valid sample for immunohistochemistry (IHC) and ISH techniques when prepared by careful selection in a manner that is representative of the corresponding whole sections.^28,41-43^ However, it is acceptable that selecting some areas of tissue for TMA block construction diminishes the value of representation of whole tissue section.

 In our study using TMA technology, we transferred 2 cores with 1 mm diameter from donor blocks into TMA blocks. In a study by Truong et al, which was performed on TMA blocks using EBER ISH on three tissue cores with 1 mm diameter from each normal and tumor donor block, 12 out of the 235 tumors (5.1%) exhibited positive EBV expression.^14^ In a study by Birkman et al, two 1 mm cores from the center of the tumor and two from the periphery or invasive front of the tumor were transferred to TMA blocks. EBV RNA was found to be present in 17 (9.1%) out of 186 intestinal tumors, while none of the 49 diffuse tumors was EBV-positive; so, overall, 7.8% of gastric cancers were EBV-positive.^44^ In a study by Song et al, in which two 2.0-mm tissue cores were taken from representative regions of each paraffin block, EBVaGC was identified in 128 cases (11.9%) from a total of 1080 gastric cancers analyzed.^45^

 Another reason for discrepancies in different studies could be due to the subtypes of gastric cancers and their distribution. The subtype of gastric cancer which is most associated with EBV is medullary carcinoma or carcinoma with lymphoid stroma. The pooled prevalence of EBV positivity among the lymphoepithelioma-like gastric carcinoma cases in a systematic review was 90.5%. The lymphoepithelioma-like gastric carcinoma cases comprised between 0.9% and 15% of the studies in this review.^17^

 We found higher frequency of EBV positivity in medullary carcinoma and younger age which are similar to the study by Yanagi et alin the Japanese population.^46^ In their study, 80 (7.1%) of gastric cancers were positive for EBER-1 ISH. They also found that carcinoma with lymphoid stroma was found in 3.8% of all of their tumors, and 60.5% of these tumors were EBV-positive.

 In a study by Ojima et al, EBVspecific RNA was detected in 83 (20.1%) of gastric cancers and concordant to our results, most of these EBVaGCs (60 cases) were gastric carcinoma with lymphoid stroma.^47^

 In the study by Noh et al, which was performed on paraffin blocks of 956 cases of gastric carcinoma by EBER-1 ISH, the EBV positive cases were 65 (6.8%) and similar to our study, these cases were also more associated with the gastric carcinoma with lymphoid stroma morphology.^48^

 In research by Uner et al, 40 gastric carcinomas with lymphoid rich stroma were included among 53 gastric carcinomas with insignificant glandular differentiation. These cases were also studied with IHC for MLH1, PMS2, MSH2 and MSH6, EBER ISH, PCR for microsatellite instability, and BRAF V600E mutation analysis. The results suggested that advanced gastric carcinoma with lymphoid rich stroma is related to two different groups: those with defective DNA mismatch repair (30 cases) and those that are EBV-associated (10 cases).^49^

 In a study by Kim et al, EBV-encoded RNA ISH was evaluated in 3499 surgical cases of gastric cancer.^50^ Two hundred and fourteen cases (6.1%) were EBV positive. Four cases had heterogeneous EBV positivity, and two different histological patterns correlated with EBV status. In one pattern, the EBV-positive areas were poorly differentiated tumors with increased lymphocytic infiltration. In the other pattern, the EBV-positive cells had a more infiltrative pattern, and metastases in the lymph nodes were all EBV-positive. This study also supports our study’s finding that EBVaGC is associated with tumors with prominent lymphoid rich stroma.

 Infiltrating immune cells may contribute to antitumor immunity by potentiating the eradication of infected cells.^51^ Immunohistochemical studies of medullary type gastric carcinoma showed that T-cells are distributed throughout the tumor in close contact with neoplastic cells, but lymphoid follicles nearby are mainly composed of B-lymphocytes. It seems that combined cell-mediated and humoral immunities occur in this type of gastric carcinoma, resulting in a more favorable prognosis compared with the usual type of gastric carcinoma.^52,53^ CAR-T cell therapy is an example of genetic engineering of T-cells, bringing us new opportunities for cancer therapy.^54,55^ Cancers that express viral products are attractive targets for this kind of therapy, because elimination of infected cells seems non-threatening to human health.^56^ As the presence of PD-L1 + tumor and/or tumor infiltrating immune cells is increased in tumors with EBV positivity, potentially creating PD-1 blockade vulnerability, we also suggest testing gastric cancers for PD-L1 and MSI status as predictors of response.^57^

## Conclusion

 The prevalence of EBV-associated gastric carcinoma was about 2.1% in 4 provinces of Iran, similar to most other Iranian studies which used ISH on paraffin blocks. The EBV genome was detected within the tumor cells (and not the lymphoid or stromal cells) and were more commonly found in tumors with high lymphoid stroma. These EBVaGC were seen in younger age patients and had trend toward lower pathologic stage but we did not observe any geographic preference for EBV positivity.
